# Effects of HIIT and MICT on cardiovascular function in essential hypertension: roles of inflammation, oxidative stress, and RAS axis

**DOI:** 10.3389/fcvm.2026.1835321

**Published:** 2026-07-20

**Authors:** Zhiwei Yan, Xiao Liu, Xiaofan Gao, Yaodong Guo, Hancheng Wu, Yu Gong, Yan Gao, Yuan Wang

**Affiliations:** 1 Provincial University Key Laboratory of Sport and Health Science, School of Physical Education and Sport Sciences, Fujian Normal University, Fuzhou, Fujian, China; 2 Department of Cardiology, Sun Yat-sen Memorial Hospital of Sun Yat-sen University, Guangzhou, Guangdong, China; 3 Cardiovascular and Metabolic Disorders Program, Duke-National University of Singapore Medical School, Singapore, Singapore; 4 Department of Clinical Medicine, the Second School of Clinical Medicine, Jiangxi Medical College, Nanchang University, Nanchang, Jiangxi, China; 5 Huankui Academy, Nanchang University, Nanchang, Jiangxi, China; 6 College of Kinesiology, Shenyang Sport University, Shenyang, Liaoning, China; 7 Cardiovascular Rehabilitation Center, Liaoning Jinqiu Hospital, Shenyang, Liaoning, China

**Keywords:** angiotensin, endothelial function, epinephrine, essential hypertension, high-intensity interval training

## Abstract

**Objective:**

To compare the effects of High Intensity Interval Training (HIIT) and Moderate Intensity Continuous Training (MICT) on blood pressure homeostasis, cardiovascular structure, and function, and to explore the underlying neuro-humoral regulatory mechanisms in patients with essential hypertension (EH).

**Methods:**

A total of 56 EH patients were randomly (1:1) assigned to the MICT (35 min) or HIIT (1 × 10 min with 2 min intervals) for 8 weeks, three times per week. The primary outcome was the change in flow-mediated dilation (FMD). Blood pressure, traditional cardiovascular risk factors, cardiovascular structure and function, as well as neural, specific humoral, and nonspecific humoral regulation were assessed.

**Results:**

Among 56 patients with EH who were randomized, 54 (96.4%) completed the 8-week evaluation. After an 8-week intervention, the improvement in FMD was significantly greater in the HIIT group compared with the MICT group (mean difference: 0.85% [95% CI, 0.50–1.21]). HIIT showed favored changes in nitric oxide, FMD, and endothelin-1, brachial-ankle pulse wave velocity, peak systolic velocity, corrected QT Interval, Ratio of Early Diastolic Mitral Valve Peak Flow Velocity to Early Diastolic Myocardial Velocity, angiotensin-(1–7), and C-reactive protein in EH patients (all *P* < 0.05). Both HIIT and MICT significantly improved systolic blood pressure, pulsatility index, resistive index, angiotensin II, and norepinephrine in EH patients (all *P* < 0.05), with no statistically significant differences between the two groups (all *P* > 0.05).

**Conclusion:**

Both HIIT and MICT yielded comparable effects on lowering systolic blood pressure and regulating neurohumoral factors. FMD was improved to a greater extent with HIIT, which also showed superior efficacy in arterial stiffness, hemodynamics, cardiac diastolic function, and inflammatory biomarkers.

**Clinical Trial Registration:**

https://www.chictr.org.cn, identifier ChiCTR2500097077.

## GRAPHICAL ABSTRACT

**Figure:**
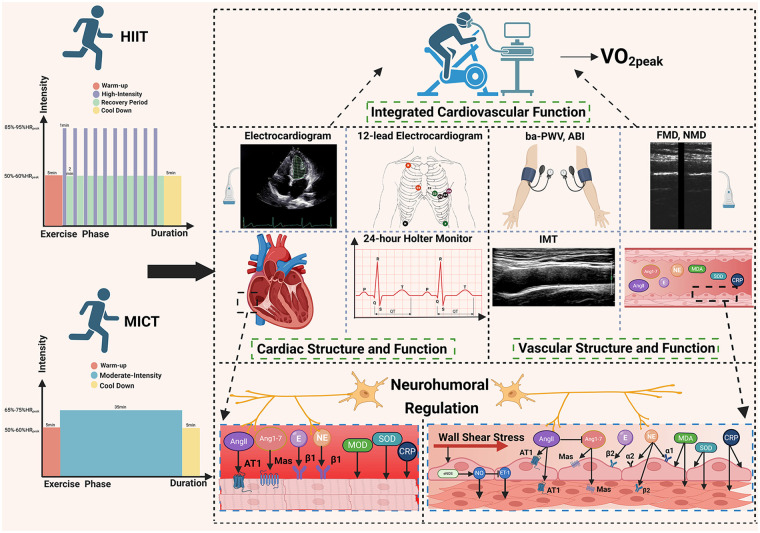

## Introduction

Essential Hypertension (EH) is a type of hypertension characterized by persistent elevation of blood pressure with no identifiable cause, accounting for approximately 90%–95% of all hypertension cases (1). If poorly controlled over the long term, EH can lead to various adverse cardiovascular and cerebrovascular events.

EH induces structural remodeling and functional impairment of the cardiovascular system, ultimately disrupting blood pressure homeostasis by affecting cardiac output (CO) and peripheral resistance (PR) (2, 3). EH affects CO by inducing chronic stress load and sympathetic nervous system excitation (2, 4). Additionally, EH contributes to increased PR by inducing vascular endothelial and smooth muscle dysfunction and enhancing arterial stiffness (5, 6).

Neuro-humoral regulatory mechanisms play a crucial role in maintaining and regulating blood pressure homeostasis. EH can activate the tension of cardiac sympathetic nerve and sympathetic vasoconstrictor fibers, leading to abnormal CO and PR (4, 7). Non-specific humoral mediators such as inflammation and oxidative stress mediate EH-induced cardiovascular injury (8, 9). In addition, EH-driven specific humoral regulatory mechanisms, including Angiotensin and catecholamines, further exacerbate EH-induced cardiovascular pathological changes (10–12). Importantly, the abnormally excited sympathetic nerve promotes the secretion of epinephrine (E) and norepinephrine (NE) and enhances the expression of angiotensin II (Ang II) (13). This neuro-humoral regulation exacerbates EH-induced cardiovascular injury. Therefore, EH induces structural damage and functional impairment of the cardiovascular system by driving complex neurohumoral mechanisms.

It is well known that moderate-intensity continuous training (MICT) can improve EH-induced cardiovascular injury through multiple neurohumoral mechanisms (14, 15). High-Intensity Interval Training (HIIT), as a time-efficient alternative to MICT, has been demonstrated to provide the same anti-hypertensive effect in EH patients (16). However, our previous research confirmed that acute MICT induced more pronounced vascular protective benefits compared to HIIT (17). Furthermore, conflicting results persist regarding the cardiovascular protective benefits of these two exercise modalities. Therefore, this study aims to compare the effects of an 8-week HIIT versus MICT regimen on blood pressure homeostasis regulation, vascular stiffness, vascular elasticity, endothelial and smooth muscle function, myocardial remodeling, myocardial contractility, and electrophysiological function in EH patients.

## Materials & methods

### Research design

**Figure 1:**
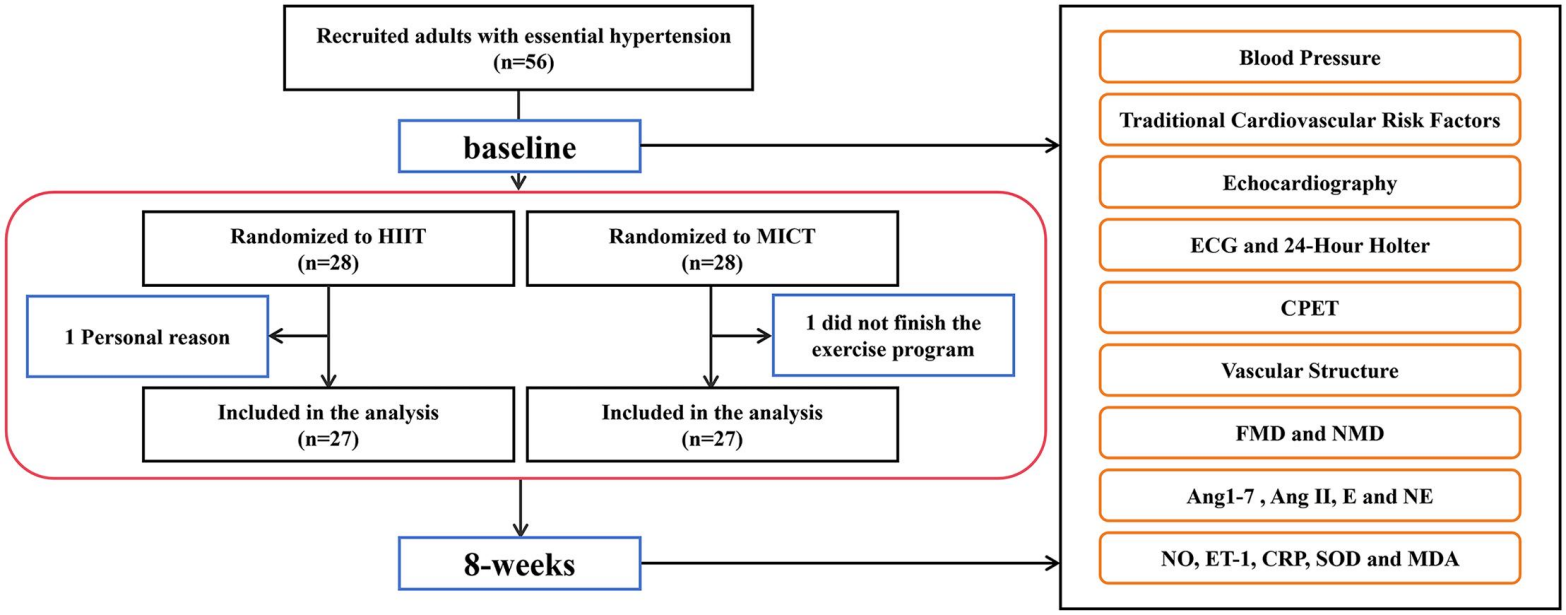
Flowchart demonstrating the randomization and group distribution of enrolled patients with essential hypertension into the HIIT group and the MICT group. HIIT, high-intensity interval training; MICT, moderate-intensity continuous training.

This was a single-blind, two-arm, parallel randomized controlled trial. Considering the potential cardiovascular protective benefits of exercise and in accordance with the experimental paradigms recommended in relevant consensus statements and guidelines, no inactive control group was included in the present study (18). An overview of the trial procedures is shown in Figure 1. The study protocol was approved by the Ethics Committee of Shenyang Sport University, adhering to the principles of the Declaration of Helsinki. The trial was registered on the Chinese Clinical Trial Registry (ChiCTR2500097077). All participants provided written informed consent prior to enrollment, and the study results were reported following the Consolidated Standards of Reporting Trials (CONSORT) guidelines.

To minimize the confounding influence of dietary factors on the experimental outcomes, participants were instructed to maintain their habitual dietary patterns throughout the entire intervention period. During the 8-week intervention, all participants were required to maintain their baseline medication regimens, and medication adjustments were meticulously documented.

### Research setting

A total of 56 EH patients completed baseline testing and were subsequently randomized in a 1:1 ratio to either the HIIT group or the MICT group using a computer-generated randomization sequence. Measurements were taken at baseline and after 8 weeks of intervention. As this study recruited hypertensive individuals from communities in Shenyang, the exercise intervention and all experimental tests were primarily conducted in Shenyang.

### Inclusion criteria

Participants were deemed eligible if they met all of the following conditions: (1) Participants aged 40 and 65 years; (2) systolic blood pressure (SBP) from 140 to 179 mmHg and diastolic blood pressure (DBP) from 90 to 109 mmHg; (3) No regular exercise habits in the 6 months prior to the study, as identified by screening with the Chinese version of the International Physical Activity Questionnaire (IPAQ); (4) Clear consciousness and voluntary participation in the exercise intervention; (5) Signed informed consent. The definition of EH is based on the Guidelines (19).

### Exclusion criteria

Participants meeting any of the following were excluded: (1) Currently participating in other scientific experiments; (2) Suffering from musculoskeletal disorders causing motor dysfunction; (3) Having cognitive impairments such as dementia; (4) Having cardiovascular or cerebrovascular diseases other than hypertension; (5) Diagnosed with cancer; (6) Having metabolic diseases such as diabetes; (7) At risk of falls; (8) secondary or malignant hypertension.

### Random assignment and blinding

Following baseline assessments, participants were randomly assigned in a 1:1 ratio to either the HIIT group or the MICT group. The randomization sequence was generated using a computer-based random number generator (Microsoft Excel) by an independent statistician who was not involved in participant recruitment or data collection, employing a simple randomization method. Allocation concealment was maintained using sequentially numbered, opaque, sealed envelopes. These envelopes were kept by a designated research assistant and were opened only after baseline assessments were completed, thereby minimizing selection bias.

This study adopted a single-blind design. After group allocation, only participants remained blinded to the assigned intervention. Blinding of the intervention staff was not feasible due to the clear differences in training mode, intensity, and operational procedures between HIIT and MICT. Personnel responsible for outcome assessment, biochemical testing, and statistical analysis remained blinded to group allocation. All data collection, sorting, and analyses were performed using anonymized coded information without any group-identifying labels. During the study, emergency partial unblinding was allowed if an acute clinical emergency occurred and the causal relationship between the intervention and unexpected adverse events could not be judged, which might affect clinical treatment decisions. Once emergency unblinding was performed, the time, cause, and operator of unblinding were fully documented, and the study monitor was notified immediately. Participants with early unblinding would terminate subsequent follow-up immediately; their data were excluded from efficacy evaluation analyses but still included in safety assessment and adverse event statistics. All participants were labeled with anonymous identification codes, and the matching list of names and codes was stored separately. After coding, group allocation data was permanently hidden in the password-protected database. Outcome assessors and statisticians only accessed coded datasets without group labels during double data entry and data review to maintain blinding.

### Intervention

This study involved 8 weeks of HIIT, and MICT performed three times weekly. Load matching between HIIT and MICT was achieved using equivalent training loads. The average Peak Oxygen Consumption (VO_2peak_) for all participants before training was 30.96 mL/kg/min. The HIIT group completed ten 1-minute exercises at 27.87 L/min (90% of VO_2peak_) and ten 2-minute exercises at 18.58 mL/kg/min (60% of VO_2peak_). The total VO_2_-time relationship for the high-intensity group was then divided by the intensity for the moderate-intensity group (650.3/18.58 = 35 min). To facilitate real-time adjustment of exercise intensity during training, the heart rate (HR) corresponding to VO_2peak_ measured by Cardiopulmonary Exercise Testing (CPET) was defined as peak heart rate (HR_peak_). Target intensity was expressed as a percentage of VO_2peak_ and converted to the equivalent percentage of HR_peak_ according to the equal-percentage principle (e.g., 60% of VO_2peak_ corresponds to 60% of HR_peak_). All participants used an HR monitor (Polar 10, Finland) during each training session. Load on the cycle ergometer was adjusted in real time during training to ensure each training segment was completed within the specified intensity range.

Additionally, during the 8-week exercise training intervention, participants were monitored using the Chinese version of the IPAQ to ensure that they did not engage in any physical activity outside the supervised training sessions. Any unsupervised physical activity was recorded for further monitoring and analysis.

### HIIT protocol

The HIIT protocol consisted of 5 min cycling warm-up (50%–60% HR_peak_), followed by 10 sets of 1 min high-intensity cycling (85%–95% HR_peak_), interspersed with 2 min of active cycling recovery (50%–60% HR_peak_) between sets, concluding with 5 min cycling cool-down (50%–60% HR_peak_). The entire session lasted approximately 40 min.

### MICT protocol

The MICT protocol consisted of 5 min cycling warm-up (50%–60% HR_peak_), followed by 35 min of moderate-intensity cycling (65%–75% HR_peak_), and concluded with 5 min cycling cool-down (50%–60% HR_peak_). The total training duration was 45 min.

Each participant received supervised training 3 times weekly for 8 weeks, with a total of 24 exercise sessions per subject. According to the available adverse event records, there were no documented cases of temporary exercise suspension due to participants’ subjective discomfort, and no serious exercise-related adverse events were reported during the intervention.

### Measurements

Blinded research assistants gathered baseline data and measurements from the participants, including their age, height, and weight. The primary outcome measure was endothelial dilation function: flow-mediated dilation (FMD). Secondary outcomes encompassed blood pressure, anthropometric parameters, cardiorespiratory function, angiotensin biomarkers, vasomotor factors, circulating catecholamines, circulating oxidative and inflammatory markers, smooth muscle dilation function, hemodynamics, vascular structure and function, and cardiac structure and function. All indicators were measured at baseline and after 8 weeks of intervention and conducted by experienced, blinded technicians. Participants were instructed to avoid strenuous exercise before testing and to abstain from consuming any food or beverages that could affect vascular responsiveness (e.g., nitrate-rich foods, alcohol, coffee) for 12 h before blood collection. 2 mL of venous blood was drawn from the elbow vein by trained medical personnel and placed into a serum separation tube. The blood sample was allowed to stand at room temperature for 30 min, then centrifuged at 4 ^°^C and 2200 g for 10 min. The serum was separated by centrifugation and stored at −80 ^°^C for subsequent analysis. The measurements of all participants at the baseline and after the intervention were performed by the same operators using the same instrument to ensure the consistency and reliability of the data. The detailed measurement methods are provided in the Supplementary Materials.

### Exercise safety and adverse events description

In accordance with the guidelines of the BMJ literature (20), strict safety protocols were adopted throughout the exercise intervention for patients with essential hypertension to guarantee participant safety and study validity. Prior to each session, all participants received routine health screening and were required to promptly report any discomfort, including dizziness, chest tightness, and fatigue. Any subjective malaise led to the immediate suspension of the exercise session. Exercise termination criteria were strictly complied with the guidelines of the BMJ literature, including angina-like symptoms, marked blood pressure lability, or other acute adverse manifestations. Once these occurred, exercise was stopped immediately, and participants were closely monitored until vital signs stabilized. All adverse events, such as musculoskeletal injuries and cardiovascular symptoms, were documented prospectively using standardized case report forms. An independent Data Safety Monitoring Board (DSMB) reviewed all events to assess severity, causal relationship with exercise, and the need for protocol modification or participant withdrawal.

### Statistical analysis

For continuous variables, normally distributed data are expressed as mean (SD). For univariate analysis between groups, the t-test is used when variances are homogeneous, and Welch's corrected t-test is applied when variances are heterogeneous. Non-normally distributed data are presented as median (interquartile range), with group comparisons performed using the Mann–Whitney U nonparametric test. Categorical data are expressed as frequencies (n, %), and intergroup differences are analyzed using the chi-square test (*χ*^2^ test). For intragroup comparisons, the paired samples t-test is used if the intragroup differences follow a normal distribution, whereas the Wilcoxon matched-pairs signed-rank test is utilized when intragroup differences are non-normally distributed. All patients were analyzed according to their randomization group. Between-group differences were analyzed using analysis of covariance (ANCOVA) adjusted for baseline values. *post-hoc* comparisons were performed using the Tukey test. *P* values derived from the Tukey test were further corrected via false discovery rate (FDR), and intention-to-treat (ITT) analysis with baseline-based missing value imputation is presented in the Supplementary Materials. To avoid over-adjustment and preserve statistical power in this small but well-balanced cohort, only the baseline value of each corresponding outcome was adjusted in the ANCOVA model. Age, BMI, and medication status were not included as additional covariates because these baseline characteristics were comparable between groups and did not confound intergroup comparisons. All data analyses were conducted using R software (version 4.3.3), with statistical significance set at *p* < 0.05. The detailed sample size calculation is provided in the Supplementary Materials.

## Results

A total of 56 patients with EH were enrolled in this study. After eligibility screening prior to randomization, all participants underwent blinded assessment of baseline indicators. Finally, 54 participants completed the 8-week exercise intervention (Figure 1). Their baseline indicators were as follows: mean age was 54 years, 25 women [46%], mean SBP was 156 mmHg, mean DBP was 84 mmHg, median body mass index (BMI) was 24.35 kg/m^2^, mean waist-to-hip ratio (WHR) was 0.85, and median FMD% was 7.20. Detailed baseline characteristics are presented in Table 1.

**Table 1:** Baseline characteristics of participants.

Characteristic	Moderate-intensity continuous training (*n* = 27)	High-intensity interval training (*n* = 27)	*p* value
Age, years	53 ± 7	54 ± 6	0.824
Sex			1.000
Female	12 (44.44)	13 (48.14)	
Male	15 (55.56)	14 (51.85)	
Duration of hypertension, years	6 (3–10)	5 (4–10)	0.931
Medication (%)	27	27	0.324
ACEI (%)	0 (0)	2 (7.41)	
ARB (%)	12 (44.44)	10 (37.04)	
Beta-blockers (%)	7 (25.93)	4 (14.81)	
Calcium antagonists (%)	8 (29.63)	11 (40.74)	
Cardiovascular risk factors			
BMI (kg/m^2^)	24.45 (22.56–25.77)	24.24 (22.24–25.79)	0.876
waist-to-hip ratio	0.85 (0.08)	0.85 (0.10)	0.951
Fat mass (kg)	17.43 (4.74)	17.80 (3.77)	0.748
Muscle mass (kg)	48.58 (42.45–54.72)	45.84 (38.12–55.30)	0.643
Body fat rate (%)	26.27 (5.32)	27.11 (3.03)	0.483
Cardiovascular Function			
SBP (mmHg)	156 (7)	156 (6)	0.951
DBP (mmHg)	84 (73–93)	84 (66–94)	0.890
PP (mmHg)	73 (12)	74 (17)	0.934
HR (bpm)	69 (10)	69 (8)	0.785
RPP (mmHg·beats/min)	10821 (1616)	10729 (1399)	0.824
CPET			
VO2_peak_ (ml/kg/min)	30.35 (25.80–33.30)	32.28 (27.17–37.02)	0.378
CEP			
QT (ms)	350.04 (28.86)	346.81 (33.32)	0.706
QTc (ms)	423.37 (23.86)	425.00 (26.44)	0.813
RMSSD (ms)	24.00 (18.00–37.45)	24.63 (17.90–38.10)	0.755
NN50	8.00 (1.00–36.00)	11.00 (2.50–41.00)	0.614
PNN50 (%)	3.00 (0.30–17.45)	0.03 (0.01–0.14)	0.001
LF (norm)	68.09 (48.34–98.44)	46.10 (39.15–67.65)	0.087
HF (norm)	31.73 (17.32–41.88)	26.40 (17.60–41.10)	0.595
LF/HF	2.50 (1.70–3.65)	2.10 (1.10–3.75)	0.396
Cardiac Structure and Function			
LVDd (cm)	4.69 (0.39)	4.71 (0.75)	0.946
IVST (cm)	0.73 (0.61–0.79)	0.70 (0.66–0.78)	0.723
LVPWT (cm)	0.64 (0.07)	0.63 (0.07)	0.814
SV (ml)	63.24 (14.08)	66.90 (13.63)	0.336
CO (L/min)	4.36 (1.15)	4.54 (0.89)	0.531
FS (%)	33.95 (3.66)	34.09 (3.55)	0.892
EF (%)	63.81 (4.14)	63.17 (3.01)	0.515
E/e′	5.81 (4.94–6.49)	5.93 (5.06–6.70)	0.672
Vascular Structure and Function			
TPR (dyn⸱s/cm⁵)	38.08 (9.38)	35.97 (8.10)	0.380
ba-PWV (m/s)	1514.00 (1405.50–1812.00)	1568.00 (1454.50–1945.00)	0.516
ABI	1.15 (1.10–1.19)	1.15 (1.12–1.20)	0.665
FMD (%)	7.50 (6.25–8.85)	6.70 (6.05–8.75)	0.604
NMD (%)	11.66 (2.33)	11.40 (2.45)	0.686
IMT (cm)	0.07 (0.07–0.08)	0.07 (0.07–0.08)	0.427
PSV (cm/s)	103.95 (83.61–123.90)	96.40 (83.00–129.99)	0.917
PI	2.08 (0.39)	2.05 (0.35)	0.774
RI	0.79 (0.75–0.83)	0.77 (0.76–0.80)	0.405
WSS (dyn/cm^2^)	28.92 (23.80–33.55)	29.32 (24.52–33.90)	0.836
NO (umol/l)	61.95 (50.93–73.72)	57.54 (54.13–70.60)	0.716
ET-1 (pg/ml)	119.92 (107.06–130.16)	114.51 (107.34–128.50)	0.719
Biological marker			
AngⅡ (pg/ml)	80.11 (10.57)	81.64 (11.55)	0.613
Ang1–7 (pg/ml)	147.41 (27.02)	147.24 (27.38)	0.982
SOD (U/mL)	45.21 (8.14)	43.39 (8.11)	0.414
MDA (nmol/mL)	5.27 (0.71)	5.29 (0.85)	0.950
CRP (mg/L)	2.86 (2.24–3.47)	2.77 (2.21–3.54)	0.616
NE (pg/mL)	341.66 (50.32)	343.31 (55.86)	0.910
E (pg/mL)	223.01 (49.48)	229.41 (51.69)	0.644

M (IQR) for non-normally distributed data, M (SD) for normally distributed data.

ABI, ankle-brachial index; ACEI, angiotensin-converting enzyme inhibitor; Ang1–7, angiotensin 1–7; AngⅡ, angiotensin Ⅱ; ARB, angiotensin II receptor blocker; baPWV, brachial-ankle pulse wave velocity; BMI, body mass index; CEP, cardiac electrophysiology; CPET, cardiopulmonary exercise test; CO, cardiac output; DBP, diastolic blood pressure; E, epinephrine; E/e′, ratio of early diastolic transmitral flow velocity to early diastolic mitral annular velocity; EF, ejection fraction; ET-1, endothelin-1; FMD, flow-mediated dilation; FS, fractional shortening; HF, high frequency; h-CRP, high-sensitivity C-reactive protein; HR, heart rate; IMT, intima-media thickness; IVST, interventricular septal thickness; LF, low frequency; LVDd, left ventricular end-diastolic diameter; LVPWT, left ventricular posterior wall thickness; MDA, malondialdehyde; NE, norepinephrine; NMD, nitroglycerin-mediated dilation; NO, nitric oxide; NN50, number of pairs of successive normal-to-normal intervals that differ by more than 50 ms; PI, pulsatility index; PNN50, percentage of NN50 intervals among all normal-to-normal intervals; PP, pulse pressure; PSV, peak systolic velocity; QT, QT interval; QTc, corrected QT interval; RI, resistivity index; RMSSD, root mean square of successive differences; RPP, rate-pressure product; SBP, systolic blood pressure; SOD, superoxide dismutase; SV, stroke volume; TPR, total peripheral resistance; VO_2peak_, peak oxygen consumption; WSS, wall shear stress.

### Primary outcome

**Figure 2:**
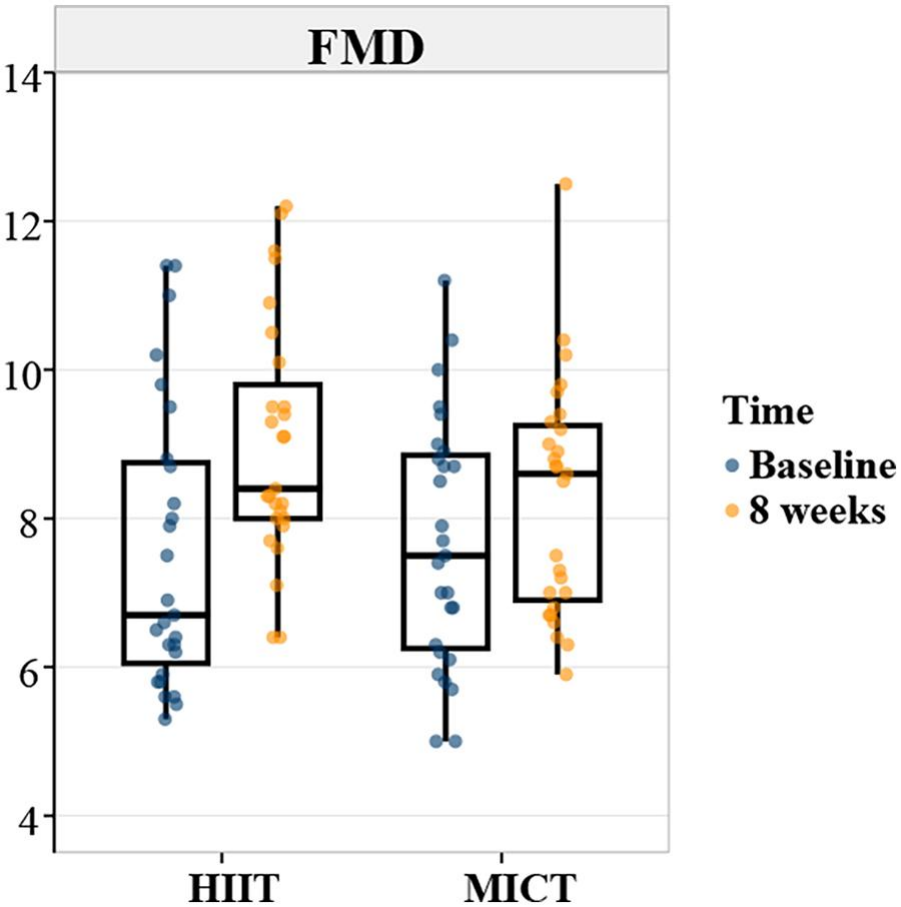
Box plots of baseline and 8-week intervention. FMD, flow-mediated dilation; HIIT, high-intensity interval training; MICT, moderate-intensity continuous training.

After 8 weeks of intervention, FMD% was significantly improved from baseline in both groups (Figure 2), with statistically significant within-group differences (HIIT: mean [SD] 1.47 [0.66]%, *P* < 0.001; MICT: 0.59 [0.78]%, *P* < 0.001). The magnitude of FMD% improvement in the HIIT group was significantly greater than that in the MICT group, with a mean difference of 0.85% [95% CI, 0.50–1.21], *P* < 0.001 (Table 2).

**Table 2:** Primary and secondary end points after 8 weeks.

Outcome/variable	MICT				HIIT				HIIT vs. MICT	
	Mean(SD)			*P* value	Mean(SD)			*P* value	Mean(SD)	*P* value
	Baseline	8 weeks	Difference		Baseline	8 weeks	Difference		Difference (95%CI)	
Primary outcome										
FMD (%)	7.67 (1.67)	8.26 (1.57)	0.59 (0.78)	<0.001	7.55 (1.92)	9.01 (1.62)	1.47 (0.66)	<0.001	0.85 (0.50 to 1.21)	<0.001
Secondary outcomes										
Cardiovascular risk factors										
BMI (kg/m^2^)	24.12 (1.99)	23.74 (2.07)	−0.38 (0.50)	<0.001	24.02 (2.22)	24.77 (2.12)	0.75 (2.87)	0.187	1.08 (0.09 to 2.06)	0.033
waist-to-hip ratio	0.85 (0.08)	0.84 (0.09)	−0.01 (0.04)	0.089	0.85 (0.10)	0.82 (0.08)	−0.03 (0.11)	0.231	−0.01 (−0.05 to 0.03)	0.508
Fat mass (kg)	17.43 (4.74)	17.45 (4.48)	0.02 (0.99)	0.908	17.80 (3.77)	18.03 (4.38)	0.22 (2.19)	0.782	0.22 (−0.71 to 1.15)	0.639
Muscle mass (kg)	48.66 (8.41)	49.09 (8.25)	0.43 (3.00)	0.463	48.69 (12.94)	50.88 (13.43)	2.19 (5.09)	0.011	1.76 (−0.52 to 4.04)	0.127
Body fat rate (%)	26.27 (5.32)	26.20 (5.27)	−0.07 (0.57)	0.525	27.11 (3.03)	26.38 (3.12)	−0.73 (0.66)	<0.001	−0.65 (−0.99 to −0.31)	<0.001
Cardiovascular Function										
SBP (mmHg)	156.37 (7.09)	153.70 (5.52)	−2.67 (6.58)	0.045	156.48 (6.05)	152.93 (5.86)	−3.56 (3.13)	<0.001	−0.84 (−3.21 to 1.53)	0.479
DBP (mmHg)	82.89 (12.10)	81.74 (11.11)	−1.15 (2.73)	0.043	82.67 (15.29)	80.67 (12.38)	−2.00 (8.09)	0.300	−0.90 (−3.73 to 1.92)	0.523
PP (mmHg)	73.48 (11.52)	71.96 (10.73)	−1.52 (6.82)	0.258	73.81 (17.18)	72.26 (13.94)	−1.56 (9.01)	0.144	0.06 (−3.69 to 3.81)	0.975
HR (bpm)	69.19 (9.79)	68.07 (5.46)	−1.11 (9.93)	0.566	68.52 (8.01)	72.41 (9.46)	3.89 (10.54)	0.137	4.48 (0.35 to 8.60)	0.034
RPP (mmHg·beats/min)	10820.63 (1616.18)	10468.44 (986.02)	−352.19 (1584.22)	0.259	10728.56 (1398.54)	11072.63 (1506.87)	344.07 (1600.73)	0.464	631.79 (−25.10 to 1288.68)	0.059
CPET										
VO2_peak_ (ml/kg/min)	30.32 (5.05)	31.00 (5.31)	0.69 (2.35)	0.141	31.60 (5.35)	33.16 (5.37)	1.56 (1.48)	<0.001	0.93 (−0.16 to 2.01)	0.092
CEP										
QT (ms)	350.04 (28.86)	350.26 (27.50)	0.22 (5.51)	0.836	346.81 (33.32)	342.96 (33.59)	−3.85 (11.89)	0.104	−4.27 (−9.28 to 0.75)	0.094
QTc (ms)	423.37 (23.86)	409.63 (20.11)	−13.74 (11.20)	<0.001	425.00 (26.44)	402.81 (24.09)	−22.19 (12.91)	<0.001	−8.07 (−13.95 to −2.20)	0.008
RMSSD (ms)	32.90 (28.56)	37.85 (22.25)	4.95 (39.95)	0.526	32.22 (21.78)	33.80 (23.60)	1.59 (31.85)	0.798	−4.12 (−16.69 to 8.45)	0.514
NN50	29.30 (43.70)	38.11 (41.51)	8.81 (61.53)	0.216	37.15 (50.97)	35.33 (45.72)	−1.81 (68.80)	0.970	−2.61 (−26.78 to 21.57)	0.830
PNN50 (%)	13.15 (20.14)	0.16 (0.26)	−12.99 (20.20)	<0.001	2.04 (8.52)	15.14 (19.73)	13.10 (22.86)	<0.001	14.25 (6.07 to 22.42)	0.001
LF (norm)	84.22 (58.83)	60.69 (42.75)	−23.54 (60.77)	0.066	62.93 (59.98)	70.31 (59.31)	7.38 (25.01)	0.137	21.82 (−0.01 to 43.64)	0.050
HF (norm)	36.37 (30.74)	47.63 (38.19)	11.25 (38.56)	0.234	31.64 (24.36)	50.06 (25.47)	18.42 (32.98)	0.007	4.08 (−13.08 to 21.24)	0.635
LF/HF	3.09 (1.99)	2.32 (2.51)	−0.77 (3.39)	0.249	3.27 (3.03)	1.69 (1.04)	−1.58 (2.89)	0.002	−0.64 (−1.70 to 0.42)	0.233
Cardiac Structure and Function										
LVDd (cm)	4.69 (0.39)	4.66 (0.37)	−0.04 (0.07)	0.003	4.71 (0.75)	4.71 (0.68)	0.00 (0.14)	0.657	0.04 (−0.01 to 0.09)	0.117
IVST (cm)	0.73 (0.13)	0.71 (0.12)	−0.01 (0.03)	0.015	0.74 (0.12)	0.73 (0.10)	−0.01 (0.05)	0.500	0.01 (−0.01 to 0.03)	0.440
LVPWT (cm)	0.64 (0.07)	0.63 (0.07)	−0.01 (0.02)	0.006	0.63 (0.07)	0.64 (0.06)	0.01 (0.03)	0.309	0.02 (0.00 to 0.03)	0.015
SV (ml)	63.24 (14.08)	63.55 (12.39)	0.32 (3.87)	0.572	66.90 (13.63)	70.91 (12.3)	4.01 (10.52)	0.009	4.67 (0.77 to 8.57)	0.020
CO (L/min)	4.36 (1.15)	4.33 (0.89)	−0.03 (0.67)	0.794	4.54 (0.89)	5.13 (1.14)	0.59 (0.84)	0.001	0.68 (0.29 to 1.07)	0.001
FS (%)	33.95 (3.66)	34.29 (2.93)	0.34 (1.91)	0.118	34.09 (3.55)	34.63 (2.39)	0.54 (2.23)	0.217	0.26 (−0.59 to 1.10)	0.543
EF (%)	63.81 (4.14)	64.02 (2.49)	0.21 (2.70)	0.407	63.17 (3.01)	63.48 (2.41)	0.31 (2.19)	0.470	−0.22 (−1.14 to 0.70)	0.629
E/e′	5.92 (1.25)	5.70 (1.13)	−0.22 (0.20)	<0.001	5.97 (1.05)	5.57 (0.80)	−0.39 (0.37)	<0.001	−0.17 (−0.29 to −0.04)	0.009
Vascular Structure and Function										
TPR (dyn⸱s/cm⁵)	38.08 (9.38)	37.06 (8.18)	−1.02 (6.14)	0.394	35.97 (8.10)	31.55 (8.55)	−4.42 (5.44)	<0.001	−3.95 (−6.91 to −0.99)	0.010
ba-PWV (m/s)	1637.11 (353.78)	1584.89 (310.43)	−52.22 (104.27)	0.015	1693.37 (368.90)	1491.59 (340.77)	−201.78 (99.75)	<0.001	−142.06 (−191.86 to −92.27)	<0.001
ABI	1.13 (0.15)	1.11 (0.13)	−0.02 (0.14)	0.454	1.13 (0.12)	1.06 (0.15)	−0.08 (0.14)	0.007	−0.05 (−0.12 to 0.01)	0.104
NMD (%)	11.66 (2.33)	12.06 (2.33)	0.40 (1.21)	0.290	11.40 (2.45)	11.90 (2.28)	0.50 (0.75)	<0.001	0.07 (−0.47 to 0.60)	0.803
IMT (cm)	0.08 (0.01)	0.07 (0.01)	0.00 (0.01)	0.102	0.07 (0.01)	0.07 (0.01)	−0.01 (0.01)	0.011	0.00 (−0.01 to 0.00)	0.335
PSV (cm/s)	107.24 (30.16)	100.43 (26.48)	−6.82 (10.11)	<0.001	105.26 (29.09)	93.83 (21.93)	−11.43 (11.27)	<0.001	−5.07 (−9.65 to −0.49)	0.031
PI	2.08 (0.39)	1.99 (0.36)	−0.09 (0.18)	0.002	2.05 (0.35)	1.91 (0.38)	−0.14 (0.23)	0.002	−0.05 (−0.16 to 0.06)	0.373
RI	0.78 (0.06)	0.74 (0.08)	−0.04 (0.06)	<0.001	0.77 (0.04)	0.72 (0.05)	−0.06 (0.05)	<0.001	−0.02 (−0.05 to 0.01)	0.298
WSS (dyn/cm^2^)	29.27 (6.31)	29.96 (6.48)	0.69 (2.55)	0.170	29.38 (6.06)	31.81 (4.94)	2.43 (2.34)	<0.001	1.75 (0.50, 3.01)	0.007
NO (umol/l)	62.20 (12.21)	67.17 (11.75)	4.96 (4.51)	<0.001	60.91 (10.13)	71.75 (10.66)	10.84 (10.05)	<0.001	5.57 (1.53 to 9.60)	0.008
ET-1 (pg/ml)	118.57 (13.51)	105.71 (12.46)	−12.86 (6.05)	<0.001	116.86 (14.48)	96.35 (13.68)	−20.51 (4.46)	<0.001	−7.88 (−10.63 to −5.14)	<0.001
NO/ET-1	0.54 (0.15)	0.65 (0.16)	0.11 (0.06)	<0.001	0.53 (0.12)	0.76 (0.16)	0.23 (0.11)	<0.001	0.12 (0.07 to 0.17)	<0.001
Biological marker										
AngⅡ (pg/ml)	80.11 (10.57)	76.73 (11.55)	−3.38 (8.46)	0.002	81.64 (11.55)	77.54 (11.81)	−4.10 (3.94)	<0.001	−0.53 (−4.11 to 3.04)	0.767
Ang1–7 (pg/ml)	147.41 (27.02)	158.22 (26.53)	10.82 (10.44)	<0.001	147.24 (27.38)	183.55 (25.48)	36.31 (5.67)	<0.001	25.48 (21.05 to 29.91)	<0.001
AngⅡ/Ang1–7	0.56 (0.14)	0.50 (0.12)	−0.06 (0.06)	<0.001	0.57 (0.14)	0.43 (0.09)	−0.14 (0.06)	<0.001	−0.08 (−0.10 to −0.05)	<0.001
SOD (U/mL)	45.21 (8.14)	44.54 (7.76)	−0.67 (2.91)	0.241	43.39 (8.11)	50.99 (8.35)	7.60 (6.25)	<0.001	7.93 (5.36 to 10.50)	<0.001
MDA (nmol/mL)	5.27 (0.71)	5.00 (0.79)	−0.27 (0.94)	0.142	5.29 (0.85)	4.76 (0.74)	−0.52 (0.46)	<0.001	−0.24 (−0.60 to 0.11)	0.175
SOD/MDA	8.74 (2.06)	9.16 (2.3)	0.42 (1.83)	0.246	8.52 (2.55)	11.02 (2.76)	2.50 (1.62)	<0.001	2.05 (1.12 to 2.97)	<0.001
CRP (mg/L)	2.90 (0.74)	2.58 (0.65)	−0.32 (0.54)	0.006	2.81 (0.79)	2.15 (0.49)	−0.66 (0.58)	<0.001	−0.39 (−0.62 to −0.16)	0.001
NE (pg/mL)	341.66 (50.32)	321.08 (46.87)	−20.58 (15.32)	<0.001	343.31 (55.86)	322.93 (54.05)	−20.38 (26.37)	<0.001	0.41 (−10.86 to 11.69)	0.942
E (pg/mL)	223.01 (49.48)	215.56 (48.38)	−7.45 (9.42)	<0.001	229.41 (51.69)	213.01 (40.99)	−16.40 (14.50)	<0.001	−8.08 (−13.65 to −2.50)	0.005

Values are presented as mean (standard deviation) or mean difference with 95% confidence interval (95%CI). Within-group and between-group comparisons were performed using analysis of covariance (ANCOVA) adjusted for baseline values, followed by Tukey's post hoc test.

95%CI, 95% confidence interval; ABI, ankle-brachial index; Ang1–7, angiotensin 1–7; AngⅡ, angiotensin Ⅱ; baPWV, brachial-ankle pulse wave velocity; BMI, body mass index; CEP, cardiac electrophysiology; CPET, cardiopulmonary exercise test; CO, cardiac output; DBP, diastolic blood pressure; E, epinephrine; E/e′, ratio of early diastolic transmitral flow velocity to early diastolic mitral annular velocity; EF, ejection fraction; ET-1, endothelin-1; FMD, flow-mediated dilation; FS, fractional shortening; HF, high frequency; HIIT, High-intensity interval training; CRP, C-reactive protein; HR, heart rate; IMT, intima-media thickness; IVST, interventricular septal thickness; LF, low frequency; LVDd, left ventricular end-diastolic diameter; LVPWT, left ventricular posterior wall thickness; MDA, malondialdehyde; MICT, moderate-intensity continuous training; NE, norepinephrine; NMD, nitroglycerin-mediated dilation; NO, nitric oxide; NN50, number of pairs of successive normal-to-normal intervals that differ by more than 50 ms; PI, pulsatility index; PNN50, percentage of NN50 intervals among all normal-to-normal intervals; PP, pulse pressure; PSV, peak systolic velocity; QT, QT interval; QTc, corrected QT interval; RI, resistivity index; RMSSD, root mean square of successive differences; RPP, rate-pressure product; SBP, systolic blood pressure; SOD, superoxide dismutase; SV, stroke volume; TPR, total peripheral resistance; VO_2peak_, peak oxygen consumption; WSS, wall shear stress.

### Secondary outcomes

#### Blood pressure

After 8 weeks of intervention, SBP was significantly decreased from baseline in both groups, with statistically significant within-group differences (HIIT: mean [SD] −3.56 [3.13] mmHg, *P* < 0.001; MICT: −2.67 [6.58] mmHg, *P* = 0.045). However, there was no significant between-group difference in the magnitude of SBP reduction (*P* = 0.479). A significant within-group decrease in DBP was observed only in the MICT group (*P* = 0.043). Additionally, there was a significant between-group difference in HR changes over 8 weeks (4.48 bpm, [95% CI, 0.35–8.60], *P* = 0.034) (Table 2).

#### Cardiovascular risk factors

After 8 weeks of intervention, BMI was significantly decreased from baseline in the MICT group (*P* < 0.001), and there was a significant between-group difference in BMI changes (1.08 kg/m^2^, [95% CI, 0.09–2.06], *P* = 0.033). A significant decrease in body fat percentage and a significant increase in muscle mass were observed only in the HIIT group (both *P* < 0.05), with a significant between-group difference in body fat rate changes (*P* < 0.001) (Table 2).

#### Vascular-related indicators

After 8 weeks of intervention, brachial-ankle pulse wave velocity (ba-PWV) was significantly decreased from baseline in both groups (both *P* < 0.05), and the magnitude of ba-PWV reduction in the HIIT group was significantly greater than that in the MICT group (−142.06 cm/s, [95% CI, −191.86 to −92.27], *P* < 0.001). A significant decrease in Intima-Media Thickness (IMT) and a significant reduction in ankle-brachial index (ABI) were observed only in the HIIT group (both *P* < 0.05) (Table 2).

After 8 weeks of intervention, nitroglycerin-mediated dilation (NMD) was significantly increased from baseline in the HIIT group (*P* < 0.001) (Table 2). Regarding vasoactive factors, nitric oxide (NO) was significantly increased (both *P* < 0.001), and endothelin-1 (ET-1) was significantly decreased (both *P* < 0.001) in both groups. The magnitude of NO increase (5.57 μmol/L, [95% CI, 1.53–9.60], *P* = 0.008) and ET-1 decrease (−7.88 pg/mL, [95% CI, −10.63 to −5.14], *P* < 0.001) in the HIIT group were significantly greater than those in the MICT group. The NO/ET-1 ratio was significantly increased in both groups (both *P* < 0.001), and the magnitude of increase in the HIIT group was significantly greater than that in the MICT group (*P* < 0.001) (Table 2).

After 8 weeks of intervention, peak systolic velocity (PSV), resistive index (RI), and pulsatility index (PI) were significantly decreased from baseline in both groups (all *P* < 0.05). Total peripheral resistance (TPR) was significantly decreased, and wall shear stress (WSS) was significantly increased in the HIIT group (both *P* < 0.001), with significant between-group differences in TPR, PSV, and WSS changes (all *P* < 0.05) (Table 2).

#### Cardiac-related indicators

After 8 weeks of intervention, left ventricular end-diastolic diameter (LVDd), interventricular septal thickness (IVST), and left ventricular posterior wall thickness (LVPWT) were significantly improved from baseline in the MICT group (all *P* < 0.05). There was a significant between-group difference in LVPWT changes (0.02 cm, [95% CI, 0.00–0.03], *P* = 0.015), but no significant between-group differences were found in LVDd and IVST changes (all *P* > 0.05) (Table 2).

After 8 weeks of intervention, the ratio of early diastolic transmitral flow velocity to early diastolic mitral annular velocity (E/e′) was significantly decreased from baseline in both groups (both *P* < 0.001), and the magnitude of decrease in the HIIT group was significantly greater than that in the MICT group (−0.17, [95% CI, −0.29 to −0.04], *P* = 0.009). Stroke Volume (SV) and CO were significantly increased in the HIIT group (both *P* < 0.05), with significant between-group differences in SV and CO changes (all *P* < 0.05) (Table 2).

After 8 weeks of intervention, corrected QT interval (QTc) was significantly shortened from baseline in both groups (both *P* < 0.001), and the magnitude of QTc shortening in the HIIT group was significantly greater than that in the MICT group (−8.07 ms, [95% CI, −13.95 to −2.20], *P* = 0.008). High frequency (HF) was significantly increased, and the low frequency/high frequency (LF/HF) ratio was significantly decreased in the HIIT group (both *P* < 0.05). Notably, the percentage of normal-to-normal intervals exceeding 50 milliseconds (PNN50) was significantly decreased from baseline in the MICT group (*P* < 0.001), whereas it was significantly increased from baseline in the HIIT group (*P* < 0.001), with a significant between-group difference in PNN50 changes (*P* = 0.001) (Table 2).

#### CPET indicators

After 8 weeks of intervention, VO₂peak was significantly increased from baseline in the HIIT group (*P* < 0.001), with no significant within-group change in the MICT group (*P* = 0.141). There was no significant between-group difference in VO₂peak changes (*P* = 0.092) (Table 2).

#### Circulating markers

After 8 weeks of intervention, angiotensin 1–7 (Ang 1–7) was significantly increased in both groups (both *P* < 0.001); angiotensin II (Ang Ⅱ), C-reactive protein (CRP), NE, and E were significantly decreased in both groups (all *P* < 0.05). The magnitude of Ang 1–7 increase (25.48 pg/mL, [95% CI, 21.05–29.91], *P* < 0.001), CRP decrease (−0.39 mg/L, [95% CI, −0.62 to −0.16], *P* = 0.001), and E decrease (−8.08 pg/mL, [95% CI, −13.65 to −2.50], *P* = 0.005) in the HIIT group were significantly greater than those in the MICT group, but no significant between-group differences were found in AngⅡ and NE changes (all *P* > 0.05). Additionally, the Ang Ⅱ/Ang 1–7 ratio was significantly decreased in both groups (both *P* < 0.001), and the magnitude of decrease in the HIIT group was significantly greater than that in the MICT group (*P* < 0.001) (Table 2).

After 8 weeks of intervention, superoxide dismutase (SOD) was significantly increased, and Malondialdehyde (MDA) was significantly decreased in the HIIT group (both *P* < 0.001), with a significant between-group difference in SOD changes (7.93 U/mL, [95% CI, 5.36–10.50], *P* < 0.001). The SOD/MDA ratio was significantly increased in the HIIT group (*P* < 0.001), with a significant between-group difference in ratio changes (*P* < 0.001) (Table 2).

After FDR correction of Tukey-derived *P* values, most results remained statistically significant, whereas several nominally significant outcomes lost statistical significance. Corresponding ITT results were also shown in Supplementary Table S1 for verification. Notably, after FDR correction, the statistically significant intergroup differences in body mass index (BMI), resting heart rate (HR), and peak systolic velocity (PSV) became non-significant.

## Discussion

This study comparing the cardiovascular protective effects of HIIT and MICT in patients with EH suggests the following findings. (1) Both HIIT and MICT may exert the same anti-hypertensive effect independently of traditional cardiovascular risk factors. (2) Compared with MICT, HIIT significantly improved arterial stiffness, endothelial diastolic function, and PR in patients with EH. HIIT independently improved vascular remodeling and smooth muscle diastolic function. (3) HIIT independently enhanced the level of VO_2peak_. (4) MICT independently reduced myocardial remodeling in EH patients, while HIIT independently enhanced cardiac systolic function. Both exercise modalities improved myocardial diastolic function in EH patients, with HIIT showing greater effects. (5) Compared to MICT, HIIT mediated more pronounced neuro-humoral regulation.

The cardiovascular protective benefits of MICT are well known. Our previous studies also suggest that MICT reduces SBP levels and improves cardiovascular risk factors in EH patients (17). However, the anti-hypertensive benefits of HIIT versus MICT remain controversial. We suggest that both exercise modalities may exert equivalent anti-hypertensive effects. These results are consistent with previous studies (16). However, some studies have indicated that HIIT may exhibit a superior anti-hypertensive effect compared to MICT (21). The reasons for heterogeneous results are complex, which may be attributed to differences in exercise intensity, exercise volume, and the pathological severity of patients themselves. Although the clinical benefits of these two exercise modalities for lowering blood pressure remain controversial, the exercise-induced reduction in blood pressure may be crucial for reducing adverse cardiovascular events.

Traditional cardiovascular factors dominated by central obesity are considered important contributors to the imbalance of blood pressure homeostasis. Previous studies have shown that both HIIT and MICT reduce body weight and BMI in EH patients (22). However, current evidence regarding the effects of these two training modalities on central obesity levels remains insufficient. This study found that neither HIIT nor MICT improved the WHR or fat mass in EH patients, which may be related to the relatively short intervention duration in this study. These results suggest that exercise-mediated SBP reduction may occur independently of traditional cardiovascular risk factors.

EH-induced vascular remodeling is specifically characterized by increased vascular wall thickness and vascular stiffness, which may elevate PR and ultimately lead to increased SBP (3). Consistent with previous findings, this study suggests that both HIIT and MICT reduced arterial stiffness in EH patients, with HIIT showing a more significant effect (23). Importantly, HIIT independently improved vascular remodeling. Vascular endothelium can regulate PR to maintain blood pressure homeostasis by secreting vasoactive factors such as NO and ET-1 (24). However, EH may induce vascular endothelial dysfunction, thereby disrupting blood pressure homeostasis. We found that both HIIT and MICT restore the balance of NO and ET-1 in the blood vessels of EH patients, with HIIT demonstrating a more significant effect. Furthermore, we also obtained consistent results with the vascular active factors by measuring FMD (the gold standard indicator for endothelial function). The results suggest that HIIT significantly improved WSS independently of MICT. This seems to explain the more pronounced endothelial diastolic performance induced by HIIT, as shear stress may activate endothelial nitric oxide synthase (eNOS) activity in endothelial cells, thereby promoting NO secretion (25). Smooth muscle diastolic dysfunction is another key factor contributing to the increased PR in EH. However, the clinical evidence for exercise improving smooth muscle function in this population remains unknown at present. Surprisingly, we suggest for the first time that only HIIT may improve vascular smooth muscle diastolic function. Finally, we found that compared to MICT, HIIT may more significantly improve hemodynamics and reduce PR.

EH-induced cardiac remodeling is specifically characterized by left ventricular hypertrophy, which may further lead to diastolic dysfunction and thus disrupts blood pressure homeostasis (2). Previous evidence has shown that regular MICT significantly reduced EH-induced left ventricular remodeling (26). However, evidence regarding the effects of HIIT on cardiac remodeling in EH patients remains insufficient. The study found that MICT reduced myocardial hypertrophy in EH patients, and compared to the HIIT group, MICT can drive a more significant ventricular cavity expansion effect. This suggests that MICT may exert more comprehensive protective effects on cardiac structure in EH. This may be due to the lack of obvious myocardial remodeling in EH patients in this study, leading to a non-sensitive response to HIIT. More importantly, the more extensive hemodynamic changes induced by HIIT may increase systolic intraventricular pressure, resulting in greater wall tension and increased cardiac thickness—thereby potentially offsetting the inhibitory effect of HIIT on myocardial hypertrophy (27). Cardiac remodeling further may lead to decreased diastolic function and compensatory changes in systolic function, resulting in abnormal elevation of CO, which may further affect blood pressure homeostasis. The results showed that both exercise modalities may significantly improve myocardial diastolic function in EH patients, with HIIT demonstrating more pronounced effects. Interestingly, we found that HIIT may independently improve SV in EH patients. The efficacy of HIIT in enhancing both systolic and diastolic myocardial function in EH patients may have been corroborated by other studies (28). Notably, we did not observe any impact of HIIT on systolic myocardial performance in EH patients. This may stem from the fact that early-stage EH is more prone to diastolic dysfunction without systolic impairment, rendering aerobic exercise incapable of yielding significant improvements (29). In addition, neuromodulatory mechanisms contribute to EH-induced myocardial contractile dysfunction. EH can induce sympathetic nervous system activation, which is frequently associated with cardiac remodeling and systolic/diastolic dysfunction, thereby increasing the risk of arrhythmias (30). Therefore, through ECG and 24-hour Holter monitoring, we found that both HIIT and MICT can reduce QTc, indicating that both exercise modalities are beneficial for reducing the risk of arrhythmia. However, HIIT is more effective in reducing the risk of arrhythmia and can independently enhance vagal tone and restore autonomic nervous system balance. As a highly integrated indicator of cardiac and vascular function, VO₂_peak_ is commonly used to predict cardiovascular disease and all-cause mortality (31). Our study suggests that HIIT may independently enhance VO₂_peak_ in EH patients, likely due to the stronger post-exercise oxygen debt resulting from high-intensity stimulation and longer exercise intervals of HIIT. The HIIT-mediated increase in VO_₂peak_ among EH patients further suggests HIIT's potential systemic protective effects on the cardiovascular system.

A variety of humoral regulatory mechanisms may be involved in exercise-mediated promotion of cardiovascular health. Non-specific humoral regulation, such as oxidative stress and inflammation, is regarded as a potential pathological mechanism leading to damage in vascular endothelial cells, smooth muscle cells, and cardiomyocytes (8, 9, 32, 33). We found that both exercise modalities may exert circulatory anti-inflammatory effects, with HIIT showing a more significant effect; a significant antioxidant effect was only observed in the HIIT group. In addition, certain cardiovascular-specific humoral regulatory mechanisms participate in cardiovascular system regulation. Circulating Ang II levels are significantly elevated in EH patients. Ang II may bind to angiotensin II type 1 receptor (AT1R) on endothelial cells and smooth muscle cells, inhibiting the eNOS-NO pathway and ultimately promoting vascular contraction (11, 12). In addition, Ang II may bind to AT1R on cardiomyocytes, inducing excessive production of ROS to exacerbate oxidative stress, thereby potentially promoting myocardial hypertrophy and further triggering cardiac dysfunction and electrophysiological remodeling (10). Our study found that both exercise modalities may downregulate Ang II levels. As an antagonistic regulatory peptide of Ang II, Ang 1–7 may bind to Mas receptors on endothelial cells, smooth muscle cells, and cardiomyocytes, activating the eNOS-NO pathway and exerting anti-inflammatory and antioxidant effects, ultimately promoting vasodilation and inhibiting myocardial hypertrophy (34–36). Our research suggests that both exercise modalities may elevate Ang 1–7 levels and reset the balance of the angiotensin axis, with HIIT demonstrating a more pronounced effect. Previous studies have shown that angiotensin-converting enzyme 2 (ACE2) can directly catalyze the hydrolysis of Ang II to generate Ang-(1–7) (38). Although direct ACE2 quantification was not performed in the present observational study, the changes in Ang II and Ang-(1–7) suggest that the stronger shear stress induced by HIIT may upregulate the expression of ACE2, thereby promoting the conversion of Ang II to Ang-(1–7) (37). Future studies may further include ACE2 quantification to clarify its role in exercise-mediated improvement of angiotensin axis balance in EH patients. In addition, E and NE are also considered to be one of the specific humoral regulatory mechanisms involved in vascular regulation. Excessive E in EH may reduce the sensitivity of beta-2 adrenergic receptor (β2-AR) on endothelial cells, thereby decreasing NO synthesis and bioavailability and ultimately leading to vasomotor dysfunction (39). Both E and NE may bind to the beta-1 adrenergic receptor (*β*₁-AR) on cardiomyocytes to increase HR and myocardial contractility. However, sustained moderately elevated levels of E and NE in EH patients may lead to excessive β1-AR stimulation, which may promote adverse cardiac remodeling and cause myocardial dysfunction (40). Studies have shown that both exercise modalities may downregulate E levels, with HIIT showing a more significant downregulating effect on E. In addition, we found that HIIT significantly reduced NE levels. The reduction in NE levels may relatively weaken the vasoconstrictive effects mediated by *α*1-AR and *α*2-AR and enhance the sensitivity of β2-AR in vascular smooth muscle, ultimately potentially improving vascular smooth muscle diastolic function (41). We speculate that the significant reduction in E and NE levels in EH mediated by HIIT may potentially alleviate myocardial remodeling and diastolic dysfunction by promoting β1-AR resensitization. Notably, HIIT and MICT exhibited distinct regulatory characteristics. In the MICT group, pNN50 declined, and NMD remained unchanged, whereas Ang II level and Ang II/Ang-(1–7) ratio were favorably modulated. These findings suggest that the cardiovascular benefits of MICT may not be predominantly manifested in cardiac autonomic regulation or endothelial vasodilator function. Instead, MICT may exert vascular protection mainly by regulating the renin–angiotensin axis, and eliciting anti-fibrotic and anti-inflammatory effects. The neuro-humoral regulation may play a crucial role in the Ang II/Ang I-7 balance and in the release of E and NE. The sympathetic nervous system has been confirmed to potentially promote the release of E and NE and stimulate renin secretion through β1-AR, thereby upregulating Ang II (13). We found that the HIIT group may reduce sympathetic tone and restore autonomic nervous balance. This may be the fundamental reason for the more significant balance of Ang II/Ang 1–7 and the reduction of E and NE in the HIIT group.

In summary, our observational study suggests that both HIIT and MICT exerted blood pressure homeostasis and cardiovascular protective benefits in EH patients. Compared with MICT, HIIT showed superior efficacy in improving arterial stiffness, endothelial dilation function, hemodynamics, SV, and myocardial diastolic function. Notably, MICT independently reduced myocardial remodeling, while HIIT independently improves cardiopulmonary function, vascular remodeling, and smooth muscle diastolic function. These differences may be related to distinct hemodynamic changes, non-specific anti-inflammatory and antioxidant humoral regulation, specific resetting of the balance between Ang II and Ang 1–7, and downregulation of E and NE, and potential modulation of the autonomic nervous system mediated by the two exercise modalities. Our results only demonstrate observational correlations rather than definitive causal mechanisms; further cellular and animal studies are warranted to elucidate the specific molecular signaling pathways.

### Limitations

This study has several limitations. First, due to the limited sample size, some indicators may have shown only trend-level changes. In addition, the limited sample size was insufficient to support sex-stratified analyses with adequate statistical power. Therefore, these findings need to be further verified in studies with larger sample sizes. Second, this study investigated the potential protective effects of exercise by measuring circulating humoral regulatory factors, yet it lacked organ-specific mechanistic analyses. Further studies at the animal or cellular level are warranted to elucidate the underlying specific mechanisms. Third, all EH patients in this study were from the Asian population, resulting in a relatively homogeneous sample structure. Thus, the study results cannot be generalized to all populations. In addition, this study only included an 8-week intervention without long-term follow-up, making it impossible to determine whether the benefits are sustainable or reversible after training cessation. Future studies should incorporate long-term assessments. Finally, the lack of ACE2 quantification limits further verification of the Ang II-to-Ang-(1–7) conversion mechanism. Given the potential cardiovascular protective benefits of exercise and in line with the experimental paradigms recommended by relevant consensus statements and guidelines, a no-treatment control group was not included in the present study.

## Conclusions

Both HIIT and MICT can exert a comparable anti-hypertensive effect independently of traditional cardiovascular risk factors. However, compared with MICT, HIIT may tend to more markedly improve vascular remodeling, endothelial and smooth muscle vasodilatory function, myocardial function, potentially by inducing more extensive hemodynamic changes, enhancing nonspecific anti-inflammatory and antioxidant effects, resetting the balance between Ang II and Ang 1–7, suppressing E and NE levels, and restoring autonomic nervous system balance.

## Data Availability

The data that support the findings of this study are available from the corresponding author upon reasonable request, subject to applicable ethical and privacy restrictions.
