# Supplementary material for: Effects of HIIT and MICT on cardiovascular function in essential hypertension: roles of inflammation, oxidative stress, and RAS axis
Source: Front Cardiovasc Med. 2026 Jul 20;13:1835321. doi: 10.3389/fcvm.2026.1835321 (PMC13429827; doi:10.3389/fcvm.2026.1835321)
Supplement: Supplementary file 1 [file Datasheet1.docx]

Effects of HIIT and MICT on cardiovascular function in essential hypertension: roles of inflammation, oxidative stress, and RAS axis

Running title: Modes of exercise in hypertension

Zhiwei Yan^1#^, Xiao Liu^2,3#^, Xiaofan Gao^4#^, Yaodong Guo^1^, Hancheng Wu^1^, Yu Gong^5^, Yan Gao^6^, Yuan Wang^7*^

1. Provincial University Key Laboratory of Sport and Health Science, School of Physical Education and Sport Sciences, Fujian Normal University, Fuzhou, Fujian, China
2. Department of Cardiology, Sun Yat-sen Memorial Hospital of Sun Yat-sen University, Guangzhou, Guangdong, China
3. Cardiovascular and Metabolic Disorders Program, Duke-National University of Singapore Medical School, Singapore, Singapore
4. Department of Clinical Medicine, The Second School of Clinical Medicine, Jiangxi Medical College, Nanchang University, Nanchang, Jiangxi, China
5. Huankui Academy, Nanchang University, Nanchang, Jiangxi, China
6. College of Kinesiology, Shenyang Sport University, Shenyang, Liaoning, China
7. Cardiovascular Rehabilitation Center, Liaoning Jinqiu Hospital, Liaoning Shenyang, China

#These authors contributed equally to this work and were considered co-first authors.

Correspondence:

Yuan Wang, Cardiovascular Rehabilitation Center, Liaoning Jinqiu Hospital, Liaoning Shenyang 110016, China. E-mail: [wangyuan0109@sina.com](mailto:wangyuan0109@sina.com)

**SUPPLEMENTAL METHODS**

Primary Outcome Measures

Flow-Mediated Dilation

This study used a vascular ultrasound system (UNEX EF38G, UNEX, Japan) to measure FMD, which is considered the gold standard for noninvasive assessment of vascular endothelial function[1]. The specific measurement procedures and precautions should be strictly implemented in accordance with the established research protocol and the latest authoritative guidelines[1, 2]. FMD was calculated as:

$$\text{FMD\%=}\frac{\left[ \left( \text{peak post-hyperemic diameter-basal arterial diameter} \right) \right]}{\text{basal arterial diameter}}\text{×100\%}$$

Each participant marked the test site with a marker pen and photographed it for consistent repositioning across assessments.

**Secondary Outcome Measures**

**Blood Pressure**

We measured the blood pressure levels of the participants using the automated blood pressure machine (Omron HEM-3, Omron, Japan) that had been rigorously calibrated and complied with international blood pressure measurement standards. The primary test indices included SBP, DBP, PP, and RPP. Specifically, RPP was calculated:

$$\text{RPP=SBP×HR}$$

Each blood pressure measurement was carried out three times, and the average value of the latter two measurements was selected as the final test result.

Cardiovascular risk factors

This study measured the body composition, BMI, and WHR of participants using a body composition analyzer (InBody 770, InBody Co, Korea), a height gauge, and a tape measure. Before measurement, participants stood barefoot in light clothing, holding electrodes with both hands and remaining stationary. The device automatically recorded weight, fat-free mass, fat mass, muscle mass, and subcutaneous fat percentage. Height was subsequently measured using a stadiometer with a precision of 0.1 cm, and BMI was calculated as weight in kilograms divided by the square of height in meters. Waist circumference (horizontal circumference at the mid-axillary line, midway between the lower edge of the costal margin and the iliac crest) and hip circumference (at the fullest part of the hips) were measured using a soft tape measure at standard body landmarks, and WHR was calculated as waist circumference in centimeters divided by hip circumference in centimeters.

**Nitroglycerin-Mediated Dilation**

The same vascular ultrasound system (UNEX EF38G, UNEX, Japan) used for FMD was employed to assess vascular smooth muscle function through NMD. Participation preparation, measurement positioning, brachial artery imaging, and probe fixation procedures were consistent with FMD. After obtaining baseline arterial diameter, 0.4 mg nitroglycerin was administered sublingually following standardized procedures to induce vascular smooth muscle relaxation. When the arterial dilation response reached a plateau, the diameter at this point was recorded. The NMD calculation formula is:

$$\text{NMD\%=}\left( \frac{\text{maximal post-nitroglycerin diameter-basal arterial diameter}}{\text{basal arterial diameter}} \right)\text{×100\%}$$

Vascular Structure and Hemodynamics

Experienced ultrasound physicians used the Doppler ultrasound equipment of the GE (GE VIVID E95, Norway) brand to evaluate the vascular structure and hemodynamic parameters of the participants. To ensure the objectivity of the evaluation results, the ultrasound physicians conducting the test were kept unaware of the experimental groupings. During the testing process, the experienced ultrasound physicians accurately measured and recorded key parameters such as IMT of the bilateral carotid arteries, the thickness of the adventitia-media, PSV, PI, and RI. TPR was calculated using the formula[3]:

$$\text{TP}\text{R}\text{=}\frac{\text{MAP}}{\text{CO}}$$

Blood flow shear stress is calculated using the following formula[4]:

$$\text{WSS=}\frac{\text{kηV }}{\text{D}}$$

k refers to a constant reflecting the relationship between the mean flow velocity and the velocity gradient at the vessel wall, and it is commonly assigned a value of 4. Η represents blood viscosity (mPa·s⁻¹). V denotes the mean carotid blood flow velocity (cm·s⁻¹), and D refers to the arterial basal internal diameter (cm).

Pulse Wave Analysis and Ankle-Brachial Index

We utilized a pulse wave detector (P-203RPEIII, Omron, Japan) to measure the ba-PWV and the ABI to assess vascular elasticity and the degree of atherosclerosis. The specific operational procedures and precautions follow our previous research[2]. The ba-PWV calculation formula is:

$$\text{ba-PWV}\text{=}\frac{\text{La-Lb}}{\text{T}}$$

Where "La" represents the length from the heart to the ankle joint, and "Lb" represents the length from the heart to the humerus. Each limb was measured three times, and the average value was included in the subsequent analysis. ABI is calculated as the ratio of the maximum SBP at the bilateral ankles to the maximum SBP at the bilateral brachial arteries:

$$\text{ABI=}\frac{\text{max (SBP\_ankle)}}{\text{max(SBP\_brachial)}}$$

Cardiopulmonary Exercise Testing (CPET)

VO_₂peak_ was measured using a power bicycle (Monark 839E, Varberg, Sweden) and a gas analyzer (MetaMax 3B, CORTEX, Germany). The workload was applied using a graded incremental protocol: 5 min warm-up at 0 W, followed by 15-30 W increments every 1 min until voluntary exhaustion[5]. Throughout the test, participants maintained a constant cadence of approximately 60-80 rpm. Heart rate was continuously monitored, and subjective exertion levels were recorded. VO_₂peak_ was calculated as the average oxygen uptake value during the final 30 seconds of exercise. When participants met any two of the following three criteria, they were considered to have exerted maximum effort and may cease exercise: (1) Respiratory exchange ratio (RER) > 1.15; (2) Heart rate > 85% of predicted HR_max_ (Predicted HR_max_ = 208 - 0.7 × age); (3) Borg scale > 17.

**Electrocardiography (ECG)**

Experienced operators assessed the cardiac electrophysiological function of participants using a 12-lead ECG (EDAN SE-1010, China) following standardized procedures (paper speed 25 mm/s, gain 10 mm/mV). Before recording, participants rested supine in a dimly lit room for 10 min. To ensure technical ECG quality, operators individually verified clear signals in all leads, the absence of myoelectric artifacts and electromagnetic interference, and confirmed adequate electrode contact. Subsequently, QT was measured and recorded, and QTc (ms) was calculated using standard methods.

$$\text{QTc=}\frac{\text{QT}}{\sqrt{\text{RR}}}$$

Ambulatory ECG

Experienced operators assessed the autonomic nervous systems of participants using 24-hour Holter monitoring (EDAN SE-1201Pro, China). Testing and data acquisition strictly adhered to Holter monitoring standards[6]. Analytical indicators encompass both time-domain and frequency-domain metrics. Time-domain indicators include RMSSD, NN50, and pNN50. Frequency-domain indicators comprise HF (0.15 Hz - 0.4 Hz), LF (0.04 Hz -0.15 Hz), and LF/HF.

Echocardiogram (ECHO)

Routine cardiac structural and functional parameters were measured using color Doppler ultrasound (Vivid E95, GE Healthcare, USA). During the examination, subjects were placed in the left lateral decubitus position, with simultaneous ECG monitoring for synchronization. A phased-array transducer (M5S, harmonic frequency: 1.7–3.4 MHz) was utilized, and dynamic images were acquired for over 3 consecutive cardiac cycles, including the parasternal long-axis view of the left ventricle, apical four-chamber view (encompassing the apical four-chamber view focused on the right heart), apical two-chamber view, apical long-axis view, and short-axis views of the left ventricle at the basal, mid-ventricular, and apical levels.

Routine measurements were performed on the standard parasternal long-axis view of the left ventricle: LVDd, IVST, and LVPWT were measured at left ventricular end-diastole (corresponding to the peak of the R wave on the ECG) and left ventricular end-systole (corresponding to the end of the T wave on the ECG), respectively. EF was derived using Simpson's biplane method based on images from the standard apical four-chamber and two-chamber views.

Mitral valve inflow velocity parameters were obtained from the standard apical four-chamber view: the pulsed-wave Doppler sample volume was positioned at the level of the mitral valve leaflets to measure the early diastolic peak flow velocity (E). Under tissue Doppler imaging mode in the standard apical four-chamber view, the pulsed-wave Doppler sample volume was placed at two sites of the mitral annulus (the lateral wall of the left ventricle and the interventricular septal side) to measure the early diastolic peak motion velocity of the septal mitral annulus (e1) and the lateral mitral annulus (e2), respectively. The average of e1 and e2 (denoted as e') was calculated, and E/e' was further computed.

**Angiotensin**

Serum levels of Ang II and Ang1-7 were measured using ELISA in all participants. The Ang II and Ang 1-7 assay kits were purchased from Jiangsu Enzyme Exemption Industry CO., Ltd (Ang II: MM-0004H2, Ang 1-7: MM-1719H2). The analysis of the aforementioned biomarkers was conducted strictly in accordance with the instructions provided by the ELISA kit.

NO, and ET-1

Serum NO levels were measured using a microplate colorimetric assay, and ET-1 levels were determined by ELISA. The NO assay kit was purchased from Jiangsu Edison Biotechnology CO., Ltd (NO: ADS-W-N005-96), while the ET-1 assay kit was obtained from Jiangsu Enzyme Exemption Industry CO., Ltd (ET-1: MM-0998H1). The analysis of the aforementioned biomarkers was conducted strictly in accordance with the instructions provided by the ELISA kit.

E and NE

Circulating E and NE levels were measured by ELISA using commercial kits (Nanjing SenBeiJia Biotechnology Co., Ltd., China) (E: SBJ-H0965-96T; NE: SBJ-H0141-96T). The analysis of the aforementioned biomarkers was conducted strictly in accordance with the instructions provided by the ELISA kit.

Oxidative Stress and Inflammatory Markers

Serum SOD and MDA levels were measured using a microplate colorimetric assay, while CRP levels were determined using ELISA in all participants. The SOD and MDA assay kits were purchased from Jiangsu Edison Biotechnology CO., Ltd (SOD: ADS-W-KY011, MDA: ADS-W-YH002), while the CRP assay kit was obtained from Jiangsu Enzyme Exemption Industry CO., Ltd (CRP: MM-0135H1). The analysis of the aforementioned biomarkers was conducted strictly in accordance with the instructions provided by the ELISA kit.

Sample size calculation

A statistical power of 0.90 and an alpha level of 0.05 (two-sided) were used. Based on a previous study, a mean (SD) difference in the change of FMD of 3.56 (3.50) % between the HIIT and MICT groups was assumed[7]. The minimum required sample size of 22 for each group was calculated. We estimated the dropout rate to be 20%, leading to an overall sample size of 56 patients. The sample size was determined using PASS 15 software (NCSS Corp.).

**References**

1. Thijssen, D.H.J., et al., *Expert consensus and evidence-based recommendations for the assessment of flow-mediated dilation in humans.* Eur Heart J, 2019. **40**(30): p. 2534-2547.

2. Feng, W., et al., *Acute effects of high-intensity interval exercise and moderate-intensity continuous training on arterial stiffness and endothelial function in hypertension: A crossover trial.* Sci Rep, 2025. **15**(1): p. 37086.

3. Park, C., et al., *Elevated blood pressure in adolescence is attributable to a combination of elevated cardiac output and total peripheral resistance: evidence against a hyperkinetic state.* Hypertension, 2018. **72**(5): p. 1103-1108.

4. He, H., et al., *The effects of HIIT compared to MICT on endothelial function and hemodynamics in postmenopausal females.* J Sci Med Sport, 2022. **25**(5): p. 364-371.

5. Hinton, T., et al., *Carotid chemoreflex control of blood pressure at rest and during exercise in young-onset hypertension.* J Physiol, 2025. **603**(8): p. 2313-2332.

6. Steinberg, J.S., et al., *2017 ISHNE-HRS expert consensus statement on ambulatory ECG and external cardiac monitoring/telemetry.* Heart Rhythm, 2017. **14**(7): p. e55-e96.

7. Molmen-Hansen, H.E., et al., *Aerobic interval training reduces blood pressure and improves myocardial function in hypertensive patients.* Eur J Prev Cardiol, 2012. **19**(2): p. 151-60.

**Table S1 Comparisons of study outcomes between the HIIT and MICT groups**

|  | **Per-protocol analysis** | |  | **Intention-to-treat analysis** |  |
| --- | --- | --- | --- | --- | --- |
|  | **HIIT vs. MICT** | |  | **HIIT vs. MICT** |  |
|  | **Mean(SD)** | **P value** | **P value**  **(FDR correction)** | **Mean(SD)** | **P value** |
|  | **Difference (95%CI)** |  |  | **Difference (95%CI)** |  |
| **Primary outcome** |  |  |  |  |  |
| FMD (%) | 0.85 (0.50 to 1.21) | <0.001 | <0.001 | 0.83 (0.47, 1.19) | <0.001 |
| **Secondary outcomes** | | | |  |  |
| **Cardiovascular risk factors** | | | |  |  |
| BMI (kg/m²) | 1.08 (0.09 to 2.06) | 0.033 | 0.072 | 1.05 (0.10, 2.00) | 0.031 |
| waist-to-hip ratio | -0.01 (-0.05 to 0.03) | 0.508 | 0.641 | -0.01 (-0.05, 0.03) | 0.526 |
| Fat mass (kg) | 0.22 (-0.71 to 1.15) | 0.639 | 0.712 | 0.21 (-0.69, 1.11) | 0.638 |
| Muscle mass (kg) | 1.76 (-0.52 to 4.04) | 0.127 | 0.208 | 1.69 (-0.51, 3.89) | 0.129 |
| Body fat rate (%) | -0.65 (-0.99 to -0.31) | <0.001 | 0.002 | -0.62 (-0.96, -0.29) | <0.001 |
| **Cardiovascular Function** | | | |  |  |
| SBP (mmHg) | -0.84 (-3.21 to 1.53) | 0.479 | 0.634 | -0.86 (-3.16, 1.44) | 0.458 |
| DBP (mmHg) | -0.90 (-3.73 to 1.92) | 0.523 | 0.641 | -1.00 (-3.73, 1.72) | 0.464 |
| PP (mmHg) | 0.06 (-3.69 to 3.81) | 0.975 | 0.975 | -0.76 (-4.57, 3.04) | 0.689 |
| HR (bpm) | 4.48 (0.35 to 8.60) | 0.034 | 0.072 | 4.88 (0.83, 8.93) | 0.019 |
| RPP (mmHg·beats/min) | 631.79 (-25.10 to 1288.68) | 0.059 | 0.116 | 553.24 (-91.52, 1198.00) | 0.091 |
| **CPET** | | | |  |  |
| VO2_peak_ (ml/kg/min) | 0.93 (-0.16 to 2.01) | 0.092 | 0.170 | 0.90 (-0.15, 1.95) | 0.093 |
| **CEP** | | | |  |  |
| QT (ms) | -4.27 (-9.28 to 0.75) | 0.094 | 0.170 | -4.14 (-8.98, 0.70) | 0.092 |
| QTc (ms) | -8.07 (-13.95 to -2.20) | 0.008 | 0.025 | -7.80 (-13.89, -1.70) | 0.013 |
| RMSSD (ms) | -4.12 (-16.69 to 8.45) | 0.514 | 0.641 | -3.84 (-15.95, 8.26) | 0.527 |
| NN50 | -2.61 (-26.78 to 21.57) | 0.830 | 0.865 | -2.63 (-25.91, 20.65) | 0.822 |
| PNN50 (%) | 14.25 (6.07 to 22.42) | 0.001 | 0.005 | 13.42 (5.44, 21.39) | 0.001 |
| LF (norm) | 21.82 (-0.01 to 43.64) | 0.050 | 0.102 | 21.16 (0.04, 42.28) | 0.050 |
| HF (norm) | 4.08 (-13.08 to 21.24) | 0.635 | 0.712 | 4.00 (-12.61, 20.62) | 0.631 |
| LF/HF | -0.64 (-1.70 to 0.42) | 0.233 | 0.357 | -0.62 (-1.65, 0.41) | 0.232 |
| **Cardiac Structure and Function** | | | |  |  |
| LVDd (cm) | 0.04 (-0.01 to 0.09) | 0.117 | 0.197 | 0.04 (-0.01, 0.09) | 0.106 |
| IVST (cm) | 0.01 (-0.01 to 0.03) | 0.440 | 0.599 | 0.01 (-0.01, 0.02) | 0.394 |
| LVPWT (cm) | 0.02 (0.00 to 0.03) | 0.015 | 0.038 | 0.02 (0.00, 0.03) | 0.014 |
| SV (ml) | 4.67 (0.77 to 8.57) | 0.020 | 0.049 | 4.66 (0.89, 8.43) | 0.016 |
| CO (L/min) | 0.68 (0.29 to 1.07) | 0.001 | 0.005 | 0.66 (0.28, 1.04) | 0.001 |
| FS (%) | 0.26 (-0.59 to 1.10) | 0.543 | 0.649 | 0.26 (-0.55, 1.07) | 0.517 |
| EF (%) | -0.22 (-1.14 to 0.70) | 0.629 | 0.712 | -0.22 (-1.10, 0.67) | 0.626 |
| E/e' | -0.17 (-0.29 to -0.04) | 0.009 | 0.025 | -0.16 (-0.28, -0.04) | 0.011 |
| **Vascular Structure and Function** | | | |  |  |
| TPR (dyn⸱s/cm⁵) | -3.95 (-6.91 to -0.99) | 0.010 | 0.027 | -3.84 (-6.71, -0.97) | 0.01 |
| ba-PWV (m/s) | -142.06 (-191.86 to -92.27) | <0.001 | <0.001 | -137.08 (-187.57, -86.59) | <0.001 |
| ABI | -0.05 (-0.12 to 0.01) | 0.104 | 0.181 | -0.05 (-0.12, 0.01) | 0.099 |
| NMD (%) | 0.07 (-0.47 to 0.60) | 0.803 | 0.856 | 0.07 (-0.45, 0.58) | 0.797 |
| IMT (cm) | 0.00 (-0.01 to 0.00) | 0.335 | 0.483 | 0.00 (-0.01 to 0.00) | 0.372 |
| PSV (cm/s) | -5.07 (-9.65 to -0.49) | 0.031 | 0.072 | -4.96 (-9.46, -0.46) | 0.032 |
| PI | -0.05 (-0.16 to 0.06) | 0.373 | 0.522 | -0.05 (-0.15, 0.06) | 0.391 |
| RI | -0.02 (-0.05 to 0.01) | 0.298 | 0.443 | -0.02 (-0.04, 0.01) | 0.305 |
| WSS (dyn/cm²) | 1.75 (0.50, 3.01) | 0.007 | 0.025 | 1.70 (0.48, 2.92) | 0.007 |
| NO (umol/l) | 5.57 (1.53 to 9.60) | 0.008 | 0.025 | 5.38 (1.38, 9.37) | 0.009 |
| ET-1 (pg/ml) | -7.88 (-10.63 to -5.14) | <0.001 | <0.001 | -7.60 (-10.8, -4.40) | <0.001 |
| NO/ET-1 | 0.12 (0.07 to 0.17) | <0.001 | <0.001 | 0.11 (0.06, 0.16) | <0.001 |
| **Biological marker** | | | |  |  |
| Ang Ⅱ (pg/ml) | -0.53 (-4.11 to 3.04) | 0.767 | 0.835 | -0.51 (-3.98, 2.96) | 0.769 |
| Ang 1-7 (pg/ml) | 25.48 (21.05 to 29.91) | <0.001 | <0.001 | 24.6 (19.52, 29.69) | <0.001 |
| Ang Ⅱ/Ang 1-7 | -0.08 (-0.10 to -0.05) | <0.001 | <0.001 | -0.07 (-0.10, -0.05) | <0.001 |
| SOD (U/mL) | 7.93 (5.36 to 10.50) | <0.001 | <0.001 | 7.64 (5.12, 10.16) | <0.001 |
| MDA (nmol/mL) | -0.24 (-0.60 to 0.11) | 0.175 | 0.278 | -0.24 (-0.58, 0.11) | 0.169 |
| SOD/MDA | 2.05 (1.12 to 2.97) | <0.001 | <0.001 | 1.98 (1.07, 2.89) | <0.001 |
| CRP (mg/L) | -0.39 (-0.62 to -0.16) | 0.001 | 0.001 | -0.38 (-0.61, -0.14) | 0.002 |
| NE (pg/mL) | 0.41 (-10.86 to 11.69) | 0.942 | 0.962 | 0.43 (-10.64, 11.50) | 0.939 |
| E (pg/mL) | -8.08 (-13.65 to -2.50) | 0.005 | 0.020 | -7.67 (-13.2, -2.14) | 0.007 |

**Note:** Values are expressed as mean difference with 95% confidence interval (95%CI). Unadjusted P-values were obtained by Tukey’s post hoc test. Adjusted P-values were calculated using the false discovery rate (FDR) correction for multiple comparisons; Intention-to-treat analysis was performed using analysis of covariance (ANCOVA) adjusted for baseline values.
**Abbreviations:** 95%CI, 95% confidence interval; ABI, ankle-brachial index; Ang 1-7, angiotensin 1-7; Ang Ⅱ, angiotensin Ⅱ; baPWV, brachial-ankle pulse wave velocity; BMI, body mass index; CEP, Cardiac Electrophysiology; CPET, Cardiopulmonary Exercise Test; CO, cardiac output; DBP, diastolic blood pressure; E, epinephrine; E/e, ratio of early diastolic transmitral flow velocity to early diastolic mitral annular velocity; EF, ejection fraction; ET-1, endothelin-1; FMD, flow-mediated dilation; FS, fractional shortening; HF, high frequency; HIIT, High-intensity interval training; CRP, C-reactive protein; HR, heart rate; IMT, intima-media thickness; IVST, interventricular septal thickness; LF, low frequency; LVDd, left ventricular end-diastolic diameter; LVPWT, left ventricular posterior wall thickness; MDA, malondialdehyde; MICT, Moderate-intensity continuous training; NE, norepinephrine; NMD, nitroglycerin-mediated dilation; NO, nitric oxide; NN50, number of pairs of successive normal-to-normal intervals that differ by more than 50 ms; PI, pulsatility index; PNN50, percentage of NN50 intervals among all normal-to-normal intervals; PP, pulse pressure; PSV, peak systolic velocity; QT, QT interval; QTc, corrected QT interval; RI, resistivity index; RMSSD, root mean square of successive differences; RPP, rate-pressure product; SBP, systolic blood pressure; SOD, superoxide dismutase; SV, stroke volume; TPR, total peripheral resistance; VO2_peak_, peak oxygen consumption; WSS, Wall Shear Stress.
